# Supplementary material for: Steroid hormone-induced wingless ligands tune female intestinal size in Drosophila
Source: Nat Commun. 2025 Jan 6;16:436. doi: 10.1038/s41467-024-55664-2 (PMC11704138; doi:10.1038/s41467-024-55664-2)
Supplement: Supplementary file 1 — Supplementary Information [file 41467_2024_55664_MOESM1_ESM.pdf]

Steroid hormone-induced wingless ligands set female intestinal size in *Drosophila*

Lisa Zipper, Bernat Corominas-Murtra and Tobias Reiff

Supplemental figures

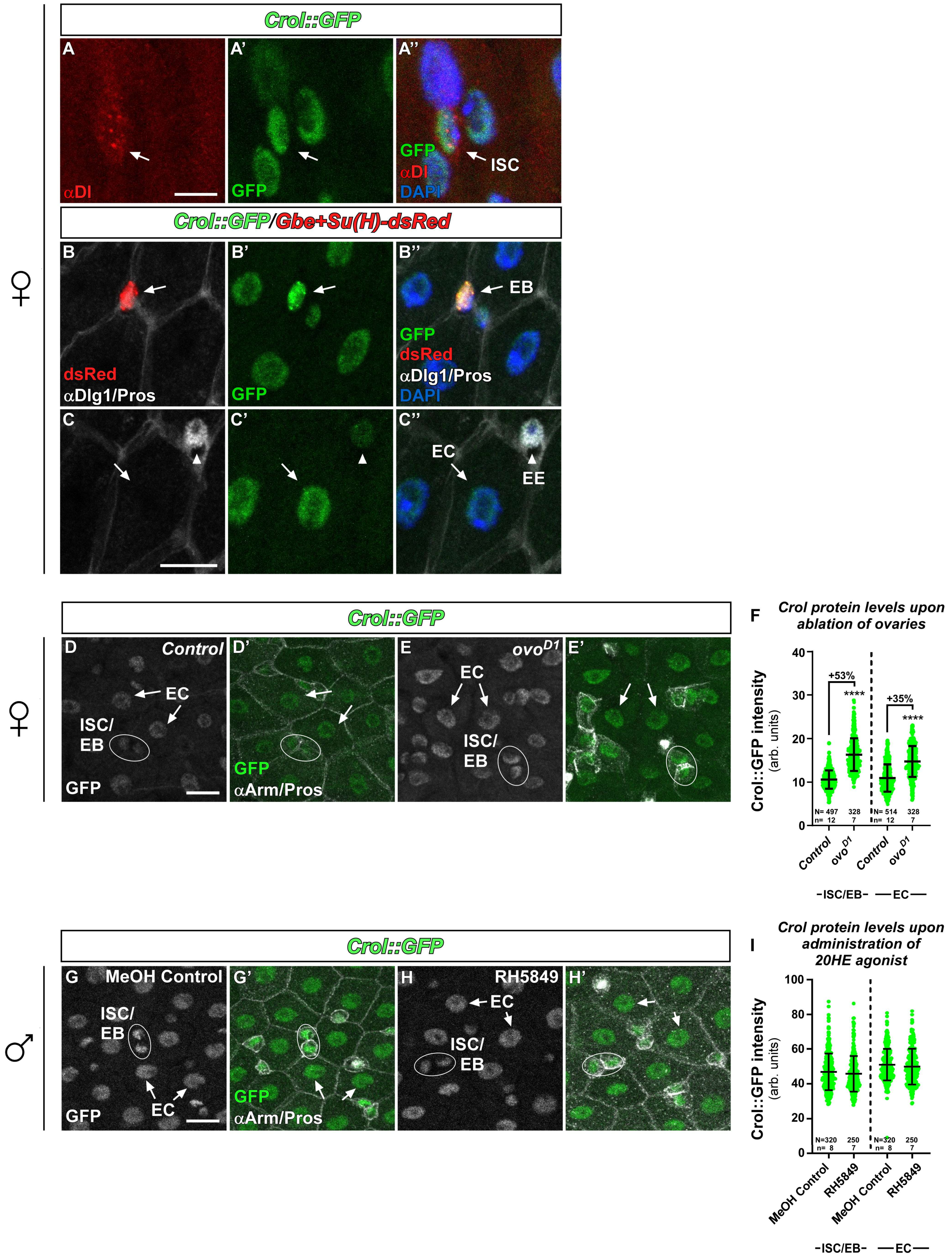

Fig.S1: Crol is expressed in the adult *Drosophila* midgut

(A-A'') Confocal images of Crol::GFP in adult midguts of MF showing localization of Crol in nuclei of ISC positive for antibody staining targeting the ISC marker Delta (αDl) and marked with white arrows. Scale bar is 5µm. (B-C'') Confocal images of Crol::GFP combined with the EB marker Gbe+Su(H)-dsRed showing Crol localization in (B-B'') dsRed positive EB marked by white arrows, and (C-C'') in epithelial EC (white arrows) stained with αDlg1 and EE (white arrowheads) stained by αPros. Scale bar is 10µm. (D-E') Confocal images showing Crol::GFP in R5 regions of mated female midguts in (D-D') w^{1118} controls compared to (E-E') heterozygous ovo^{D1} mutants. (D-E) Sole Crol::GFP signal is shown in greyscale and (D'-E') in green colour combined with antibody staining targeting Arm and Pros for identification of ISC/EB, EC and EE. Duplets of ISC/EB are outlined by white ellipses and EC are marked with white arrows. Scale bar is 10µm. (F) Quantification of Crol::GFP intensities in ISC/EB and EC in control MF compared to heterozygous ovo^{D1} mutant MF. (G-H') Confocal images showing Crol::GFP in R5 regions of adult midguts of (G-G') male flies fed with MeOH (MeOH Control) and (H-H') males fed with the 20HE agonist RH5849. (G-H) Sole Crol::GFP signal is shown in greyscale and (G'-H') in green colour combined with antibody staining targeting Arm and Pros for identification of ISC/EB, EC and EE. Duplets of ISC/EB are outlined by white ellipses and EC are marked with white arrows. Scale bar is 10µm. (I) Quantification of Crol::GFP intensities in ISC/EB and EC in males fed with MeOH compared to males fed with RH5849. (F,I) Scatter dot plots show individual values with indication of means ± SD. N and n values represent number of cells and number of biological replicas respectively. Asterisks denote significances from comparisons by two-sided Mann Whitney U tests (****$p<0.0001$). Source data are provided as a Source Data file.

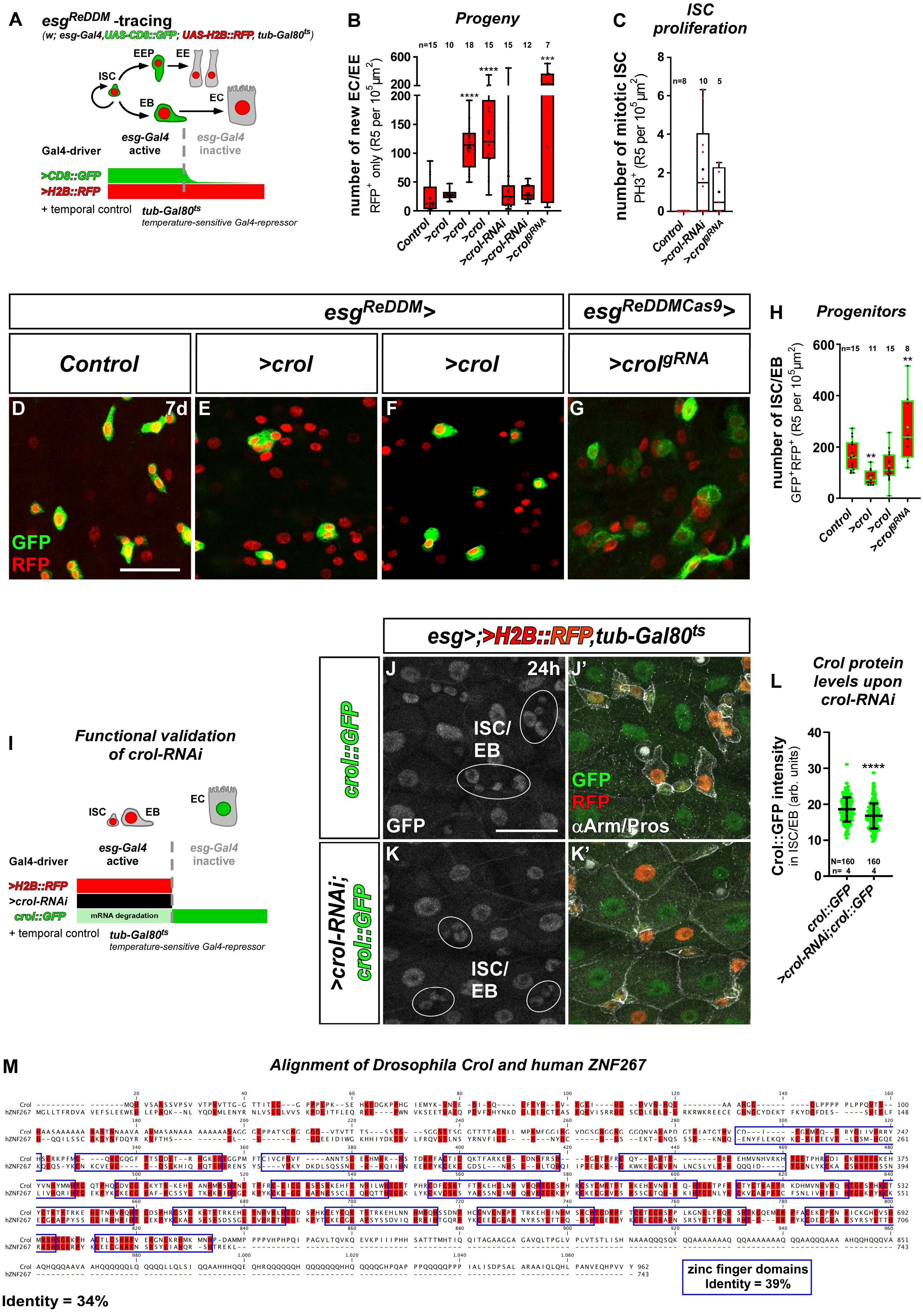

A
esgReDDM -tracing
(w; esg-Gal4,UAS-CD8::GFP; UAS-H2B::RFP, tub-Gal80ts)
ISC
EEP
EE
EB
EC
Gal4-driver
esg-Gal4 active
esg-Gal4 inactive
>CD8::GFP
>H2B::RFP
+ temporal control
tub-Gal80ts
temperature-sensitive Gal4-repressor
B
Progeny
number of new EC/EE
RFP+ only (R5 per 10^5 µm^2)
Control
>crol
>crol-RNAi
>crolgRNA
C
ISC proliferation
number of mitotic ISC
PH3+ (R5 per 10^5 µm^2)
esgReDDM>
esgReDDMCas9>
Control
>crol
>crolgRNA
D
7d
E
F
G
GFP
RFP
H
Progenitors
number of ISC/EB
GFP+RFP+ (R5 per 10^5 µm^2)
I
Functional validation of crol-RNAi
>crol-RNAi
crol::GFP
mRNA degradation
esg>;>H2B::RFP,tub-Gal80ts
J
24h
J'
ISC/ EB
αArm/Pros
K
K'
>crol-RNAi; crol::GFP
L
Crol protein levels upon crol-RNAi
Crol::GFP intensity in ISC/EB (arb. units)
crol::GFP
>crol-RNAi;crol::GFP
M
Alignment of Drosophila Crol and human ZNF267
Crol
hZNF267
zinc finger domains
Identity = 39%
Identity = 34%

Fig.S2: Crol controls ISC proliferation

(A) In *esg*^{ReDDM} tracing *esg-Gal4* double marks ISC/EB driving expression of *>CD8::GFP* (membrane, green) and *>H2B::RFP* (nuclei, red). Upon differentiation new EC/EE lose CD8::GFP, whereas nuclear H2B::RFP persists due to its long half-life. Transgene expression is timely controlled by an ubiquitously expressed Gal80^{ts} repressor (*tub-Gal80*^{ts}). (B-C) Quantification of (B) progeny (*>crol*^{gRNA}:p=0.0004) and (C) PH3^{+} mitotic active ISC upon *crol* manipulations using *esg*^{ReDDM} (B: F003414,BL58359,BL56762,BL41669,BL44643, C: BL41669). (D-G) Confocal images showing R5 regions of MF after 7d of *esg*^{ReDDM} tracing in (D) controls, (E-F) with *>crol* (BL58359,BL56762) and (G) CRISPR/Cas9 induced knockout of *crol* by *esg*^{ReDDMCas9} driven expression of guideRNAs targeting *crol* (*>crol*^{gRNA}). Scale bar is 50µm. (H) Quantification of progenitor cell numbers upon *crol* manipulations using *esg*^{ReDDM} (BL58359:p=0.0048, BL56762, *>crol*^{gRNA}:p=0.0012). (B-C,H) For Box-and-whisker plots: the center is the median, minima and maxima are 25th and 75th quartile and whiskers indicate full range of values. Individual values with ‘n’ representing biological replicas are shown by dots and means are indicated by ‘+’. Asterisks denote significances from multiple comparisons by (B) Kruskal-Wallis test and (H) One-way ANOVA (**p<0.01;***p<0.001;****p<0.0001). (I) Schematic for functional validation of *>crol-RNAi* and *crol::GFP* transgenes. *esg-Gal4* drives expression of *>H2B::RFP* and *>crol-RNAi* inducing degradation of Crol::GFP. (J-K’) Confocal images showing Crol::GFP combined with *esg >H2B::RFP* In R5 regions of MF midguts after shifting for 24h in (J-J’) controls and (K-K’) with *>crol-RNAi* (BL41669). (J-K) Sole Crol::GFP signal in greyscale and (J’-K’) combined with H2B::RFP signal and αArm/αPros staining for identification of different cell types. Duplets of ISC/EB are outlined by white ellipses. Scale bar is 20µm. (L) Crol::GFP levels in controls compared to *>crol-RNAi* (BL41669). Scatter dot plots show individual values with indication of means ± SD. N and n values represent number of cells and number of biological replicas respectively. Asterisks denote significances from unpaired two-sided t-test (****p<0.0001). Source data are provided as a Source Data file. (M) Alignment of *Drosophila* Crol and the human orthologue ZNF267 (hZNF267) indicating zinc finger domains with an identity of 39%, overall identity is 34%.

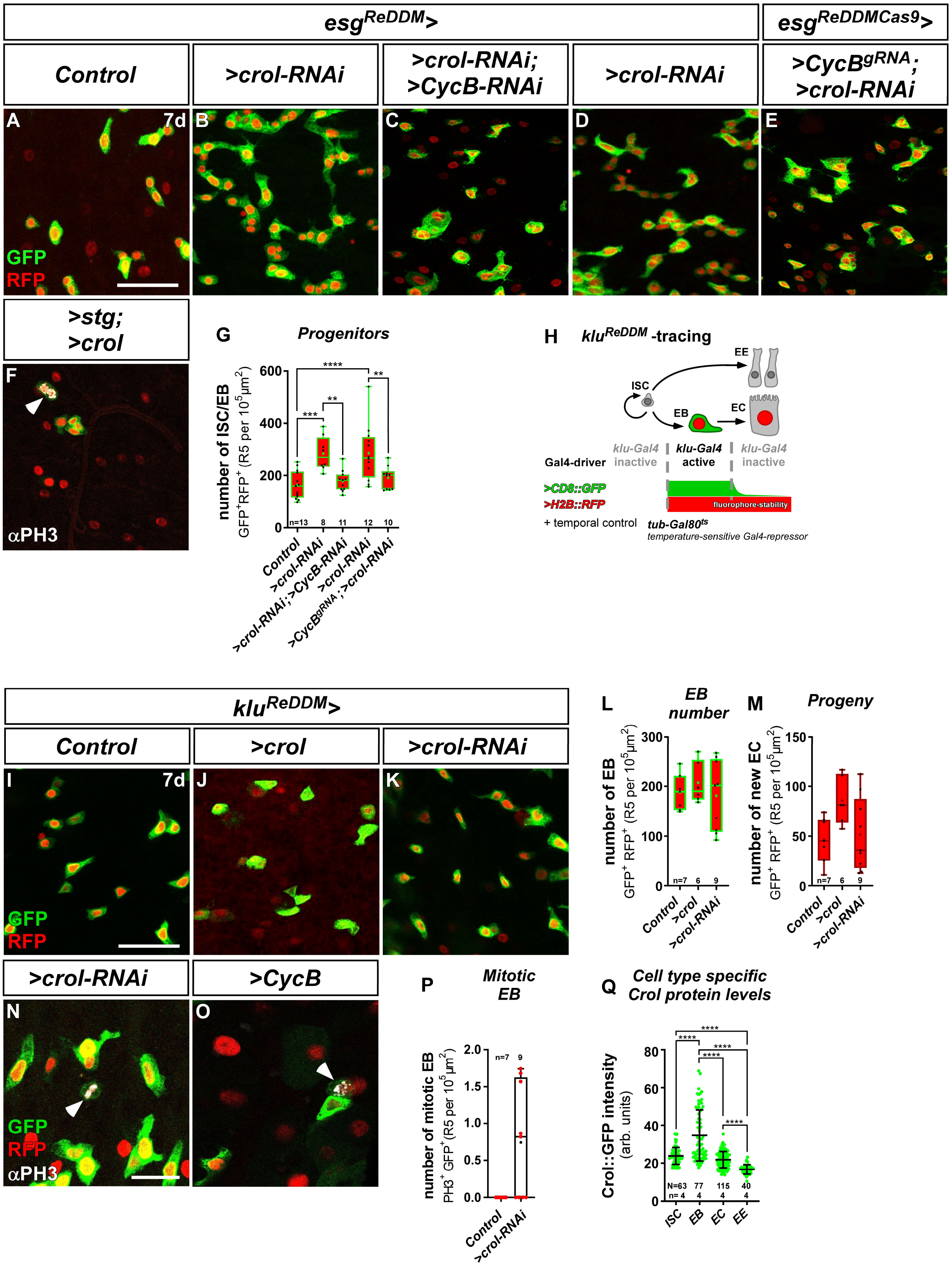

esgReDDM>
esgReDDMCas9>
Control
>crol-RNAi
>crol-RNAi; >CycB-RNAi
>crol-RNAi
>CycBgRNA; >crol-RNAi
A 7d
B
C
D
E
GFP
RFP
>stg; >crol
F
αPH3
G Progenitors
number of ISC/EB GFP+RFP+ (R5 per 10^5 µm^2)
Control
>crol-RNAi
>crol-RNAi;>CycB-RNAi
>crol-RNAi
>CycBgRNA;>crol-RNAi
H kluReDDM -tracing
ISC
EB
EE
EC
Gal4-driver
klu-Gal4 inactive
klu-Gal4 active
klu-Gal4 inactive
>CD8::GFP
>H2B::RFP
fluorophore-stability
+ temporal control
tub-Gal80ts
temperature-sensitive Gal4-repressor
kluReDDM>
Control
>crol
>crol-RNAi
I 7d
J
K
GFP
RFP
L EB number
number of EB GFP+ RFP+ (R5 per 10^5 µm^2)
M Progeny
number of new EC GFP+ RFP+ (R5 per 10^5 µm^2)
Control
>crol
>crol-RNAi
>crol-RNAi
>CycB
N
O
GFP
RFP
αPH3
P Mitotic EB
number of mitotic EB PH3+ GFP+ (R5 per 10^5 µm^2)
Control
>crol-RNAi
Q Cell type specific Crol protein levels
Crol::GFP intensity (arb. units)
ISC
EB
EC
EE

Fig.S3: Crol controls proliferation in ISC and EB by regulating CycB

(A-F) Confocal images showing R5 regions of adult female midguts after 7d of *esg*^{ReDDM} tracing in (A) controls, (B) upon expression of *>crol-RNAi* (BL41669) (C) combined with *>CycB-RNAi*, and (D) *>crol-RNAi* (BL44643) (E) combined with *>CycB*^{gRNA}, and (F) simultaneous expression of *>stg* and *>crol* (F003414) stained with an antibody targeting PH3. Scale bar is 50µm. (G) Progenitor cell numbers in combined depletions of *crol* and *CycB* using *esg*^{ReDDM} (p=0.0002;p=0.0015;p=0.0019). (H) In *klu*^{ReDDM} tracing *klu-Gal4* double marks EB driving expression of *>CD8::GFP* (membrane, green) and *>H2B::RFP* (nuclei, red). Upon differentiation new EC lose the CD8::GFP, whereas nuclear H2B::RFP persists due to its long half-life enabling tracing of EB progeny. ISC and EE are not labelled/traced by fluorophores. Transgene expression is timely controlled by an ubiquitously expressed Gal80^{ts} repressor (*tub-Gal80*^{ts}). (I-K) Confocal images showing R5 regions of MF midguts after 7d of *klu*^{ReDDM} tracing in (I) controls, (J) driving expression of *>crol* (F003414) and (K) *>crol-RNAi* (BL41669). Scale bar is 50µm. (L-M) Quantification of (L) EB numbers and (M) progeny of *klu*^{ReDDM} specific *crol* manipulations. (N-O) Confocal images showing R5 regions of female midguts after *klu*^{ReDDM} tracing and (N) expression of *>crol-RNAi* (BL41669) and (O) *>CycB*. White arrowheads point to PH3^{+} EB marked by GFP and RFP. Scale bar is 20µm. (P) Quantification of mitotic active PH3^{+} EB upon specific expression of *>crol-RNAi.* (G,L-M,P) For Box-and-whisker plots: the center is the median, minima and maxima are 25th and 75th quartile and whiskers indicate full range of values. Individual values with 'n' representing biological replicas are shown by dots and means are indicated by '+'. Asterisks denote significances from multiple comparisons by (G) One-way ANOVA and (L-M,P) Kruskal-Wallis tests (**p<0.01;***p<0.001;****p<0.0001). (Q) Crol::GFP intensities within different cell types in R5 regions of adult female midguts. Scatter dot plots show individual values with indication of means ± SD. N and n values represent number of cells and number of biological replicas respectively. Asterisks denote significances from multiple comparisons by Kruskal-Wallis test (****p<0.0001). Source data are provided as a Source Data file.

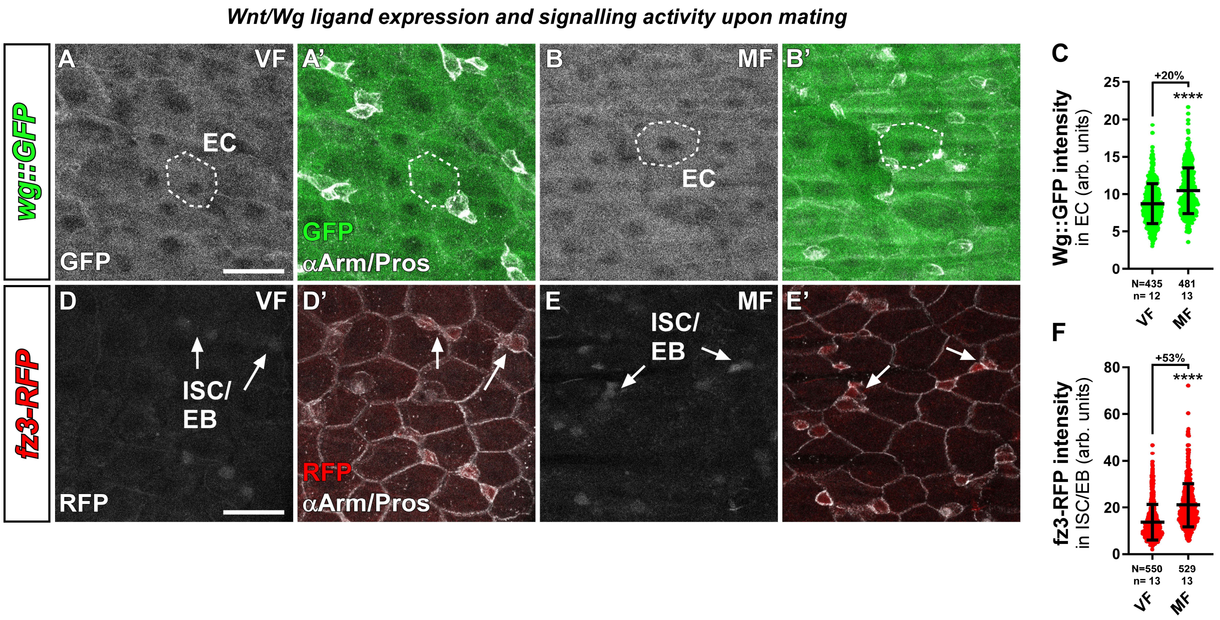

Fig.S4: Wnt/Wg activity in ISC/EB responds to mating dependent 20HE release

(A-B') Confocal images showing Wg::GFP in R5 regions of adult midguts of (A-A') VF and (B-B') MF. (A-B) Sole Wg::GFP signal is shown in greyscale and (A'-B') in green colour combined with αArm and αPros staining for identification of different cell types. Exemplary measured EC are outlined by white dashed lines. Scale bar is 20µm. (C) Quantification of Wg::GFP intensities in EC upon mating. (D-E') Confocal images showing fz3-RFP in R5 regions of adult midguts of (D-D') VF and (E-E') MF. (D-E) Sole fz3-RFP signal is shown in greyscale and (D'-E') in red colour combined with αArm and αPros staining for identification of different cell types. Exemplary measured ISC/EB are marked by white arrows. (F) Quantification of fz3-RFP intensities in ISC/EB upon mating. (C,F) Scatter dot plots show individual values with indication of means ± SD. N and n values represent number of cells and number of biological replicas respectively. Asterisks denote significances from two-sided Mann Whitney U tests (****$p<0.0001$). Fold changes are shown in percentages. Source data are provided as a Source Data file.

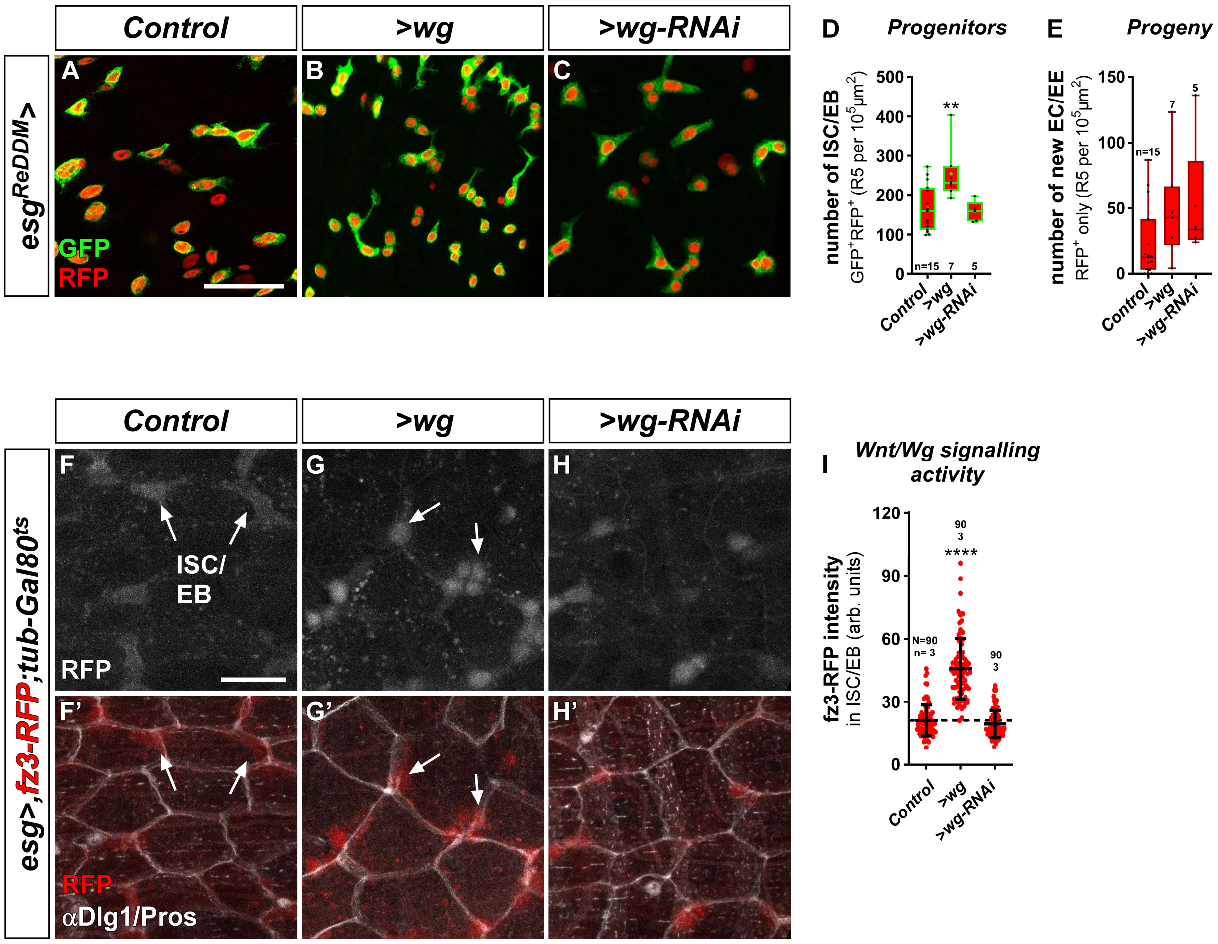

Fig.S5: Wg depletion in ISC/EB is not altering Wnt/Wg signalling activity

(A-C) Confocal images showing R5 regions of adult female midguts after seven days of *esg*^{ReDDM} tracing in (A) controls, upon (B) OE, and (C) KD of *wg*. Scale bar is 50µm. (D-E) Quantification of (D) progenitor cell numbers (p=0.0068) and (E) progeny upon ISC/EB specific manipulations of *wg.* For Box-and-whisker plots: the center is the median, minima and maxima are 25th and 75th quartile and whiskers indicate full range of values. Individual values with 'n' representing biological replicas are shown by dots and means are indicated by '+'. Asterisks denote significances from multiple comparisons by Kruskal-Wallis tests (**p<0.01). (F-H') Confocal images showing fz3-RFP signal in R5 regions of adult female midguts combined with *esg-Gal4* in (F-F') controls, (G-G') driving expression of *>wg* and (H-H') *>wg-RNAi*. (F-H) Sole fz3-RFP signal is shown in greyscale and (F'-H') in red colour combined with αDlg1 and αPros staining for identification of different cell types. Exemplary measured ISC/EB are marked by white arrows. (I) Quantification of fz3-RFP levels in ISC/EB upon *esg-Gal4* specific *>wg* and *>wg-RNAi.* Scatter dot plots show individual values with indication of means ± SD. N and n values represent number of cells and number of biological replicas respectively. Asterisks denote significances from two-sided Mann Whitney U test (****p<0.0001). Source data are provided as a Source Data file.

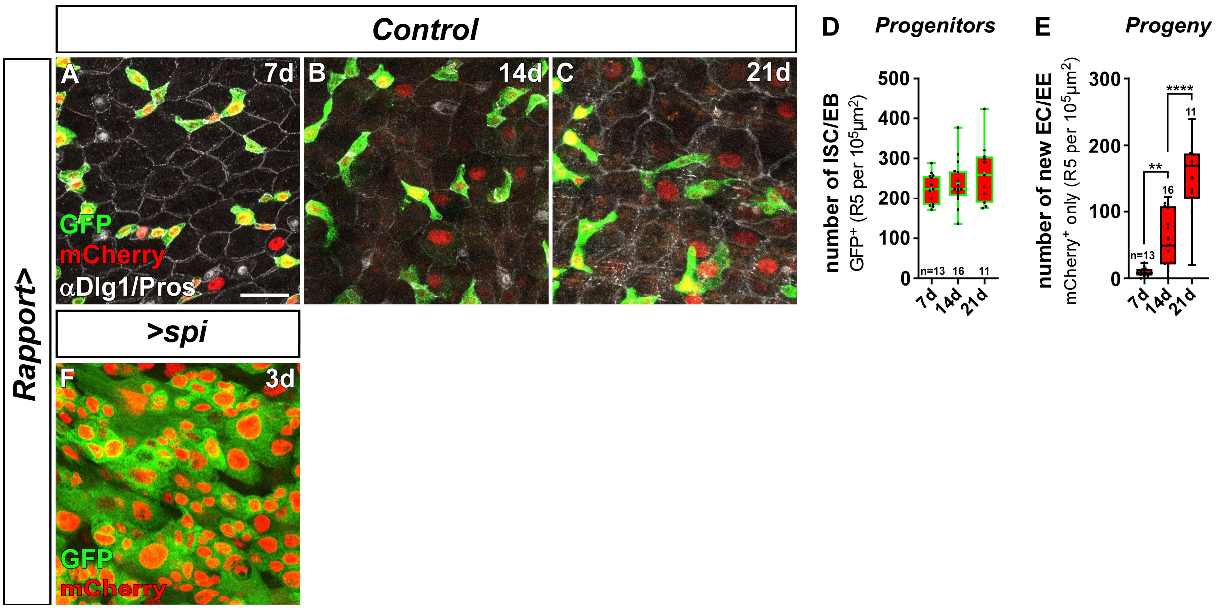

Fig.S6: Rapport tracing shows incremental tissue renewal over three weeks

(A-C) Confocal images showing Rapport tracing in R5 regions of adult female control midguts after (A) seven (7d), (B) 14 (14d), and (C) 21 days (21d) of tracing. ISC/EB are double labelled by GFP and mCherry, whereas their progeny are labelled by mCherry only. EC and EE are additionally marked by αDlg1 and αPros staining. Scale bar is 20µm. (D-E) Quantifications of (D) progenitor cell numbers and (E) progeny (p=0.0023) in control midguts upon seven, 14 and 21 days (7d, 14d, 21d) of tracing. For Box-and-whisker plots: the center is the median, minima and maxima are 25th and 75th quartile and whiskers indicate full range of values. Individual values with 'n' representing biological replicas are shown by dots and means are indicated by '+'. Asterisks denote significances from multiple comparisons by One-way ANOVA (**p<0.01;****p<0.0001). (F) Confocal image of Rapport driven expression of *UAS-spitz* (>*spi*) in the R5 region of an adult female midgut after three days (3d) of tracing. Source data are provided as a Source Data file.

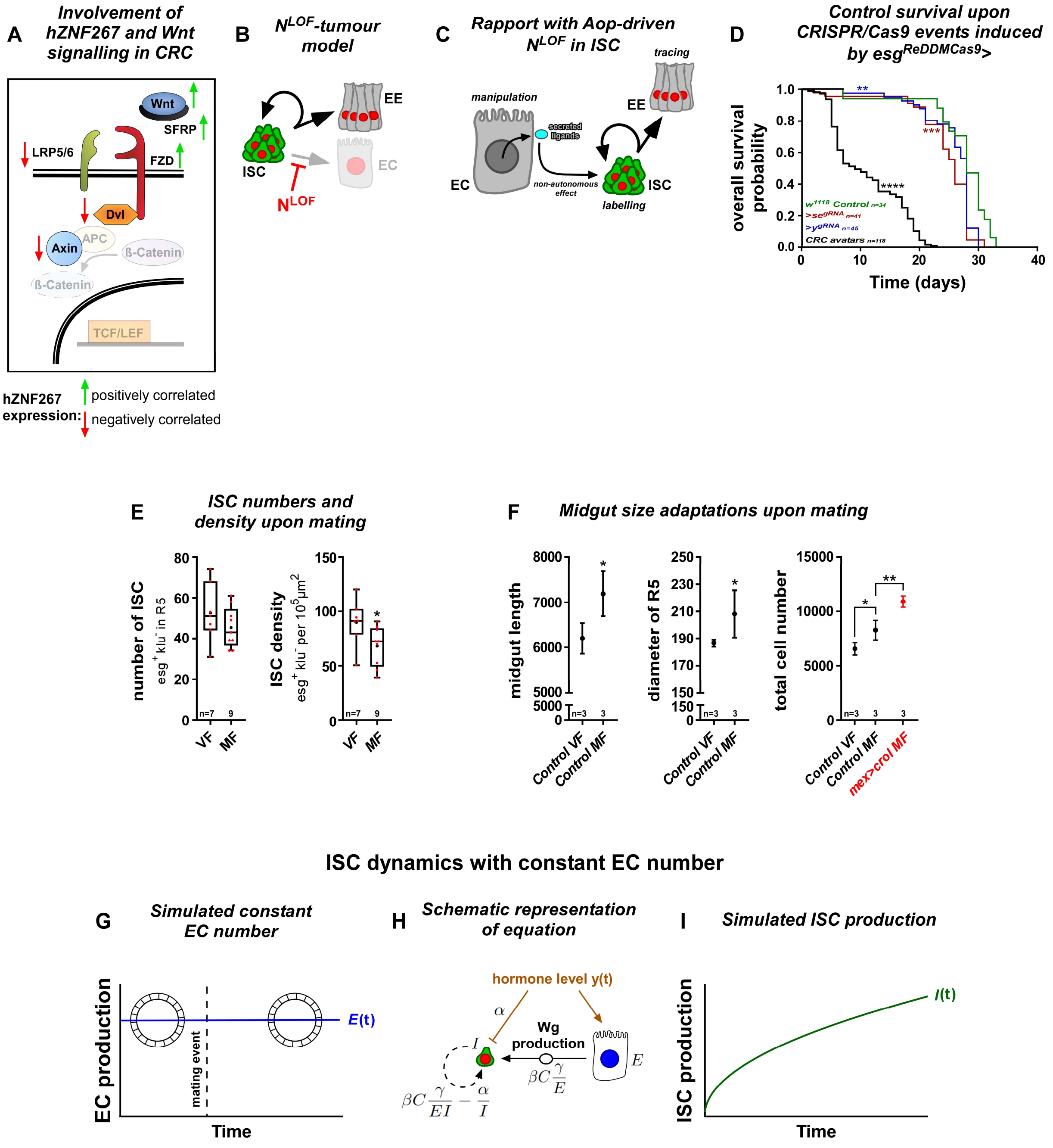
A
Involvement of hZNF267 and Wnt signalling in CRC
Wnt
SFRP
LRP5/6
FZD
Dvl
APC
Axin
ß-Catenin
ß-Catenin
TCF/LEF
hZNF267 expression:
positively correlated
negatively correlated
B
N^LOF-tumour model
EE
ISC
EC
N^LOF
C
Rapport with Aop-driven N^LOF in ISC
tracing
manipulation
EE
secreted ligands
EC
non-autonomous effect
ISC
labelling
D
Control survival upon CRISPR/Cas9 events induced by esg^ReDDMCas9>
overall survival probability
Time (days)
w^1118 Control n=34
>se^gRNA n=41
>y^gRNA n=45
CRC avatars n=118
**
***
****
E
ISC numbers and density upon mating
number of ISC
esg+ klu- in R5
ISC density
esg+ klu- per 10^5 µm^2
VF
MF
n=7
9
F
Midgut size adaptations upon mating
midgut length
diameter of R5
total cell number
Control VF
Control MF
mex>crol MF
n=3
3
ISC dynamics with constant EC number
G
Simulated constant EC number
EC production
mating event
E(t)
Time
H
Schematic representation of equation
hormone level y(t)
Wg production
I
E
I
Simulated ISC production
ISC production
I(t)
Time

Fig.S7: Additional data for tumour models and mathematical modelling

(A) Schematic of Wnt/Wg signalling pathway in humans. Marked are pathway components that positively (green arrows) and negatively (red arrows) correlate with enhanced hZNF267 expression in CRC. (B) Schematic of the N^{LOF} tumour model blocking EC differentiation thereby inducing the formation of ISC and EE tumours. (C) Schematic of *Rapport* combined with N^{LOF} by expression of *Aop-N-RNAi*. (D) Kaplan-Meier estimation of survival in $esg^{ReDDMCas9}$ w^{1118} controls and induced knockout of *yellow* (*y,* p=0.0017) and *sepia* (*se,* p=0.0001) serving as 'mock'-gRNA controls compared to CRC avatars. Number of analyzed flies is indicated by 'n' and asterisks denote significances from Kaplan-Meier estimation (**p<0.01;***p<0.001;****p<0.0001). (E) Quantification of ISC numbers and density (p=0.0459) upon mating in adult female midguts with combined expression of *esg>>CD8::GFP* and *klu>H2B::RFP* enabling identification ISC and EB. ISC numbers (absolute numbers, left) in R5 and ISC (ISC per area, right). For Box-and-whisker plots: the center is the median, minima and maxima are 25th and 75th quartile and whiskers indicate full range of values. Individual values with 'n' representing biological replicas are shown by dots and means are indicated by '+'. (F) Quantification of midgut size adaptations (p=0.0462;p=0.0156;p=0.0211;p=0.0031) upon mating in female flies of *Rapport* controls and expression of *>crol* (F003414). Midgut size adaptations encompassing entire midgut length (left), diameter of R5 (centre) and total cell number of entire midguts (right). Graphs show means ± SD. Number of biological replicas is indicated by 'n' values and asterisks denote significances from comparisons by (F,G: left and center) unpaired two-sided t-tests and multiple comparison by (E,G: right) One-way ANOVA (*p<0.05;**p<0.01). Source data are provided as a Source Data file. (H-J) Schematics of ISC dynamics with constant EC number depicted by (H) a function $E(t)$ visualizing constant EC number and intestinal size depicted by gut sections, (I) Schematic representation of equation visualizing the divergent effect of 20HE hormone on ISC (I) and EC (E) showing that ISC proliferation increases with $\beta c \frac{\gamma}{EI} - \frac{\alpha}{I}$, and (J) a function $I(t)$showing ISC proliferation over time.

Supplemental material for the mathematical model

Introduction

We construct i) a mean-field equation for the number of EC to demonstrate that the opposing roles of 20HE hormone lead to a stable population of EC and that the increase in hormone production projects into an increase of organ size. ii) a mean-field system of equations demonstrating the above claims considering a more realistic scenario where the differentiation process from ISC to EC is not happening instantaneously, thereby incorporating a transient state cell population, and iii) a mean field equation for the number of ISC in the case where differentiation is blocked, to qualitatively predict the evolution of the amount of ISC in this pathological scenario. We consider gradient diffusion and degradation of Wg ligands. In all cases, we assume that number of EC is a proxy for organ size.

For the first case, we consider the following scenario:

- 20HE activates the production of Wg in EC which, in turn, activates the production of EC from proliferation and differentiation of ISC. The production of Wg (and thus, of EC) depends on the hormone concentration (amount of hormone/cell number).

- 20HE inhibits the production of EC from differentiation of ISC at a constant rate α.

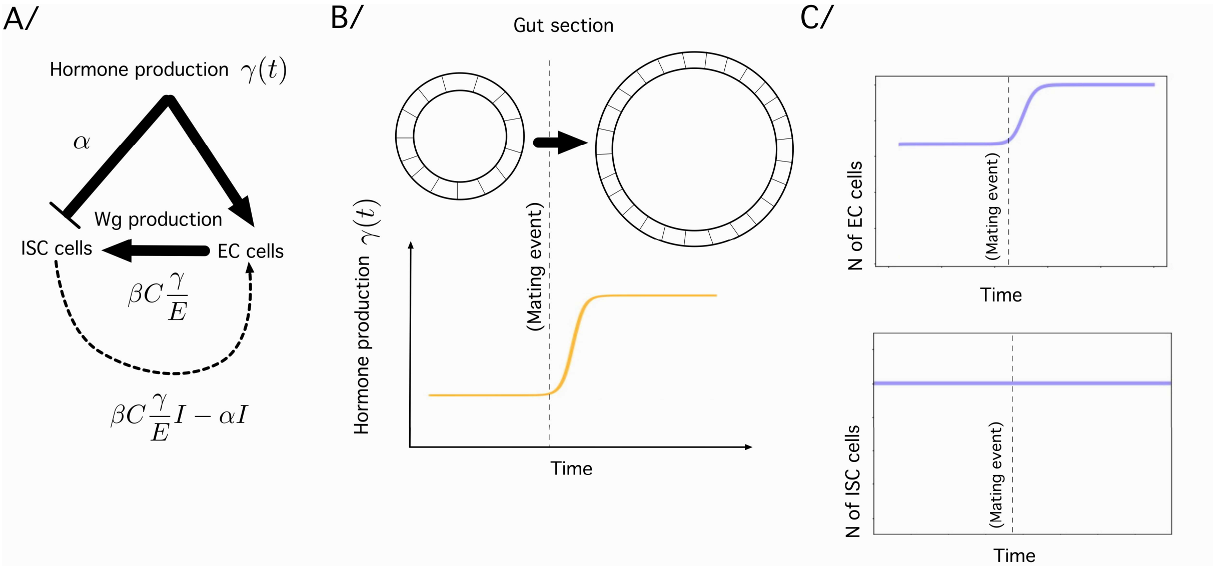

Fig.S8: **Equation showing stable population size of EC upon increased hormone levels**

A/ Construction of the dynamical equations B/ After a mating event there is an increase of midgut size (up) due to the increase in hormone production (down). C/ Evolution of the number of EC under the time evolution of the hormone production described in B/ (up). The curve is obtained integrating equation (6) with parameter γ(t) as defined in equation (1). The population of ISC remains nevertheless constant (bottom).

In Fig.S8 we provide a schematic way to understand the construction of the dynamical equations. In Fig.S9 we provide a schematic explanation of the involved terms.

Wg production and gradient

Production of Hormone

The net production of hormone γ is a function of time. At t_M a mating event triggers a sudden growth. The sudden increase of γ after a mating event can be qualitatively described by a sigmoid:

$$\gamma(t) = a\left(1 + \frac{b}{1+ce^{-(t-t_M)}}\right), \quad (1)$$

being $a, b, c > 0$ constants tuning the intensity of the hormone production. This function is plotted in orange in Fig.S8B/, down. The concentration of the hormone will be evaluated as dependent of the total amount of EC, E sharing the amount of hormone γ, i.e.:

$$\frac{\gamma}{E}.$$

Production of Wg from hormone levels

The presence of the hormone triggers the generation of Wg by EC. The amount of EC (E) will consequently represent the size of the organ, as the tissue is an epithelial (2D) tissue embedded in a cylindric geometry. We call $c(0, \gamma/E)$ the concentration of Wg at the surface of EC (E). Since the hormone levels are assumed to be constant in space, $c(0, \gamma/E)$ is assumed to be the same for all EC. We assume $c(0, \gamma/E)$ to be a monotonously growing function of γ/E. For the sake of simplicity, we will consider the dependency to be linear with proportionality constant $= 1$:

$$c(0, \gamma/E) = \frac{\gamma}{E}. \quad (2)$$

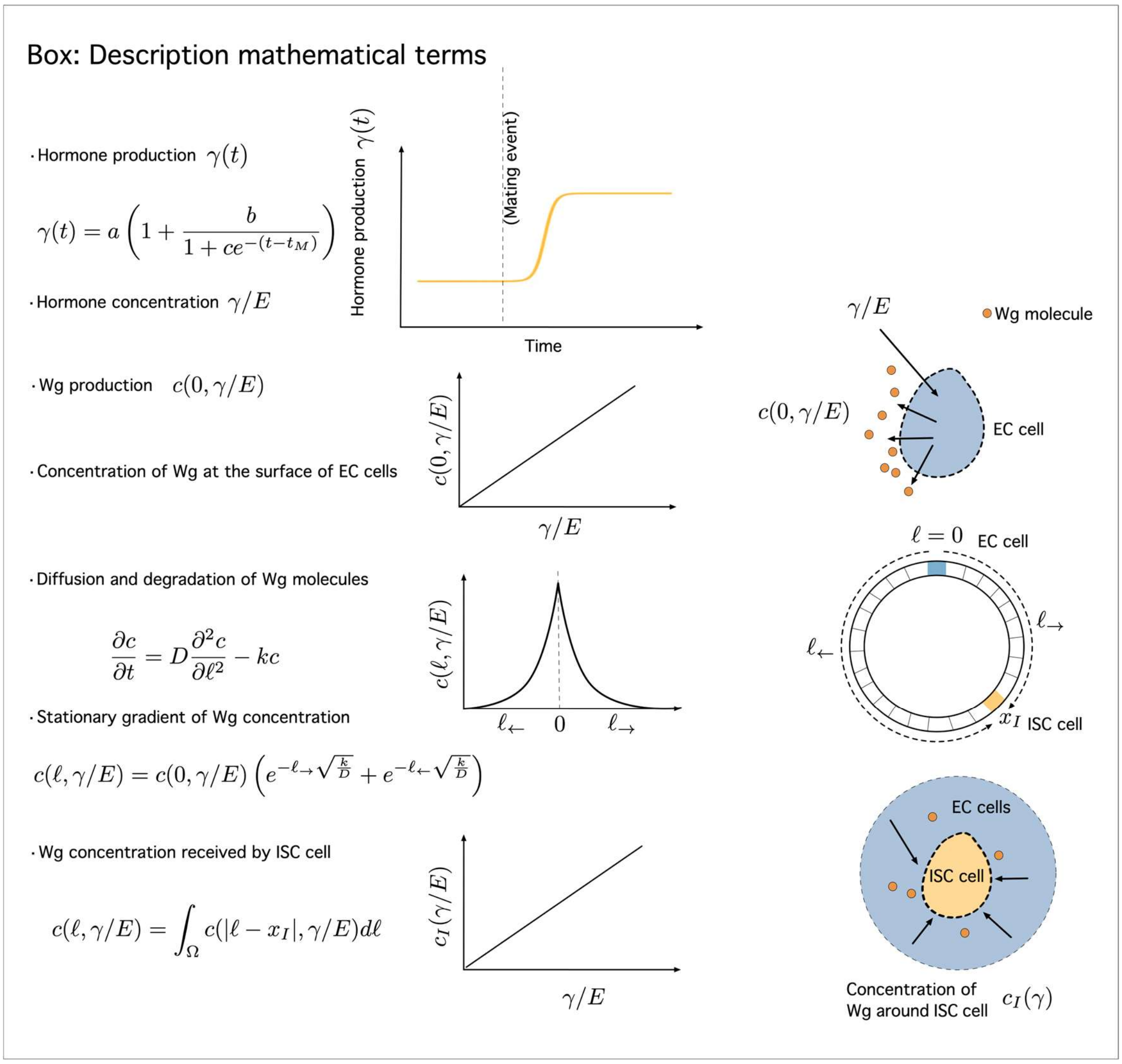

Fig.S9: **Description of the mathematical terms.**

Diffusion and degradation of Wg

We consider a section of the intestine as made of a 1D ring of cells. These cells can be either EC or ISC. Each EC occupies a fraction $\Delta\ell$ of the ring, and we approach the production of Wg with certain hormone concentration levels γ/E as:

$$\sim c(0, E/\gamma)\Delta\ell.$$

Assuming $\Delta\ell$ sufficiently small when compared with the size of the whole organ, one can consider that there is a continuum of EC with some "holes" filled by ISC. We call the set of points in the ring corresponding to EC cells Ω. Consequently, the number of EC, will be given by:

$$E = \int_\Omega d\ell\,; d\ell = Rd\theta,$$

where R is the radius of the ring, computed in units of cell diameter. Each point of Ω will produce an amount of Wg $\sim c(0, E/\gamma)d\ell$. Wg molecules will diffuse through the 1D ring with diffusion constant D and will degrade at rate k. The contribution of a single EC of the

ring to the total amount of Wg that diffuses along the ring can be grasped by the following diffusion equation with degradation [1,2]:

$$\frac{\partial c}{\partial t} = D\frac{\partial^2 c}{\partial \ell^2} - kc.$$

At the steady state, the above diffusive process will define a gradient of concentration of Wg that decays exponentially with the distance to the source ℓ:

$$c(\ell,\gamma/E) = c(0,\gamma/E)e^{-\ell\sqrt{\frac{k}{D}}}. \tag{3}$$

Since we are considering a ring, we have to take into account that the diffusion process runs in two directions from the source, clockwise and counterclockwise –see Fig.S9. Therefore, the Wg concentration at point ℓ must consider the two directions from the source:

$$c(\ell,\gamma/E) = c(\ell_{\rightarrow},\gamma/E) + c(\ell_{\leftarrow},\gamma/E),$$

where, by convention, $\ell_{\rightarrow}$ is the distance between the source and ℓ, computed clockwise (i.e.: $\ell_{\rightarrow} = \ell$); and $\ell_{\leftarrow}$ is the distance from the source and ℓ, computed counterclockwise (i.e.: $x_{\leftarrow} = 2\pi R - \ell$, where R is the radius of the ring).

Assumption: We consider that contributions to the concentration of Wg at distances beyond $2\pi R$ (the molecule traversed the whole ring already once since it departed from the source) are negligible.

Dynamical equations of the cell numbers

Concentration of Wg sensed by ISC

In the continuum approximation, at each point $\ell \in \Omega$ there will be a production of hormone $c(0,\gamma/E)$, as they are occupied by EC. The concentration of Wg at at the location of a given ISC, c_I, will be approximated by:

$$c_I(\gamma/E) = \int_{\Omega} \{c(|\ell - x_I|_{\rightarrow},\gamma/E) + c(|\ell - x_I|_{\leftarrow},\gamma/E)\}\, d\ell,$$

where x_I is the location of the ISC within the ring. According to the formal expression of $c(\ell,\gamma/E)$ given in equation (3), the above integral can be factorized as:

$$\int_{\Omega}\{\dots\}d\ell = c(0,\gamma/E)\int_{\Omega}\left\{e^{-(|\ell-x_I|_{\rightarrow})\sqrt{\frac{k}{D}}} + e^{-(|\ell-x_I|_{\leftarrow})\sqrt{\frac{k}{D}}}\right\}d\ell.$$

Interestingly, given the symmetry of the system, the above integral is constant for all ISC of the system and does not directly depend on E/γ. So, we define the constant C as:

$$C(\Omega) = \int_{\Omega}\left\{e^{-(|\ell-x_I|_{\rightarrow})\sqrt{\frac{k}{D}}} + e^{-(|\ell-x_I|_{\leftarrow})\sqrt{\frac{k}{D}}}\right\}d\ell,$$

leading, after considering the value of $c(0,\gamma/E)$ as defined in equation (2), to the following general form:

$$c_I(\gamma/E) = C(\Omega)\frac{\gamma}{E}.$$

We observe that we still have a dependency of c_I on Ω which, in turn, may depend on c_I. However, the effect of this coupling can be assumed negligible, as we detail in the following assumption.

Assumption: Due to the exponential decay of the concentration of Wg with the distance to the source, we consider that only a finite neighbourhood of EC surrounding the ISC will effectively contribute to $c_I(\gamma/E)$. In consequence, the size of Ω becomes irrelevant for the computation of c_I, as the distant EC will have a negligible effect in the concentration of Wg sensed by the ISC. Therefore, we can neglect the dependency on Ω of $C(\Omega)$:

$$C(\Omega) \Rightarrow C,$$

thereby behaving as a constant that depends only on the degradation rate k and the diffusion constant D which do not change upon changes on γ or E. In consequence, from equation (2):

$$c_I(\gamma/E) = C\tfrac{\gamma}{E}. \tag{4}$$

Dynamical equations

Collecting the above facts, and assuming that the production of EC by ISC is proportional to the amount of Wg they sense, we have a time evolution of EC with a proliferative term that reads:

$$\beta C\tfrac{\gamma}{E}I, \tag{5}$$

where I is the amount of ISC and β is a parameter that describes the strength of hormone abundance. In the main text, we set $\beta = 1$, for the sake of simplicity, as the qualitative behaviour is not affected. At the same time, the production of EC is inhibited directly by the presence of the hormone at constant rate α. Therefore, the (mean-field) equation governing the evolution of EC numbers is:

$$\frac{dE}{dt} = \beta C\tfrac{\gamma}{E}I - \alpha I. \tag{6}$$

Considering fast equilibration and, in consequence, decoupling of time scales, one can find the only equilibrium point of the above system at:

$$E^* = \beta C\tfrac{\gamma}{\alpha}. \tag{7}$$

The above fixed point is stable, as:

$$\frac{d}{dE}\left(\beta C\frac{\gamma}{E}I - \alpha I\right)\Big|_{E^*} < 0.$$

As expected:

$$\gamma(t + \Delta t) > \gamma(t) \Rightarrow E(t + \Delta t) > E(t).$$

We therefore have qualitatively proven that the combined inhibition and indirect activation of ISC proliferation proposed by our experimental data lead to a stable organ size that changes consistently with the abundance of 20HE hormone.

Non-instantaneous differentiation from ISC to EC

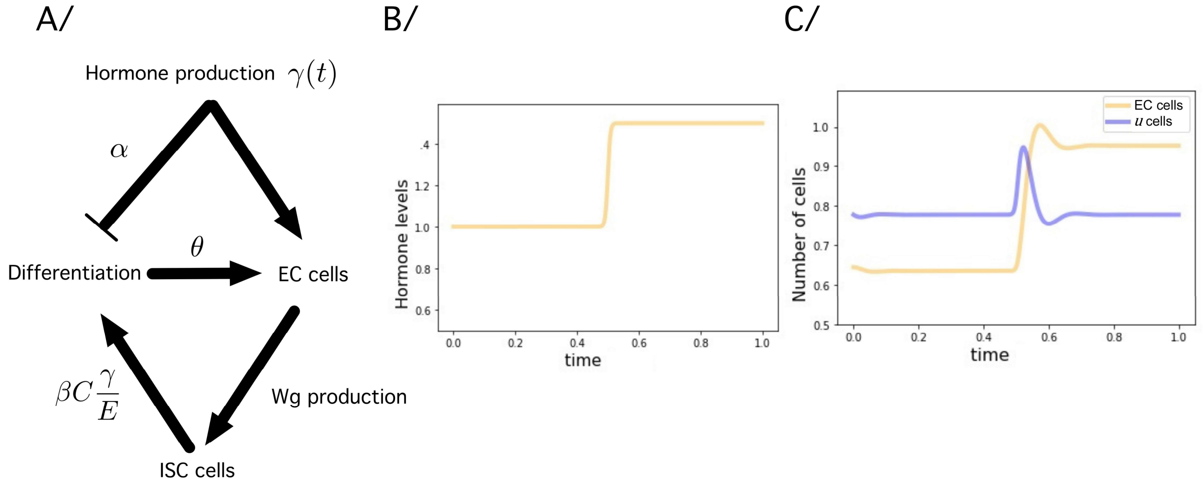

Fig.S10: **Equation showing stable population size of EC upon increased hormone levels including cells in an intermediate state**

A/ schematics of the mechanism where the transition between ISC → EC is not immediate, taking an average duration τ. Cells in process of differentiation are described by the variable u. The rates are shown by the arrows representing the transitions. According to equation (8) θ = 1/τ. The schema depicts the situation described by equations (9) and (10), i.e., case 1/. B/ A sigmoid-like pulse describing a sudden increase of 20HE hormone of 1/2 at t = 0.5. C/ Effect on the EC population and cells in transient state (u). We observe a peak in cell numbers resulting from proliferation of ISC after the increase of 20HE hormone which declines as cells are acquiring a differentiated EC identity. As predicted, the amount of EC increases with the hormone concentrations, whereas the amount of cells in transit (u) from ISC to EC goes back to its original amount despite the hormone levels increase. Integration parameters: C=10, I=10, inhibition parameter α=0.35, rate of differentiation of EC θ=0.1. Initial conditions for the integration: $u(0)$=35, $E(0)$=29.

One may consider a more realistic scenario where the differentiation from ISC into EC is not instantaneous. The natural question arises whether this affects the dynamics described so far. Here we consider that from mitosis of ISC to the full differentiation into an EC, there is a time span τ where the cell is in an intermediate state u. For this second case, we assume the following two scenarios:

- 20HE activates the production of Wg in EC which, in turn, activates the production of u cells from proliferation of ISC
- In the absence of direct inhibition of differentiation into EC, u cells last, in average, a time span τ transiting from ISC to EC
- Case 1/ 20HE inhibits the production of EC from differentiation of u cells at constant rate α

  Case 2/ 20HE inhibits the production of u cells from ISC at constant rate α

Remark: Since u cells require a finite time τ for transiting towards differentiated EC, we assume that the rate θ at which EC are produced out of u cells is:

$$\theta \approx \frac{1}{\tau}. \tag{8}$$

In Fig.S10 we provide a scheme on how the dynamics is enriched by introducing a finite time τ in the process of transition form ISC to EC. We will see that, for a large range of parameters, the dynamics is just delayed —thereby making it more realistic— but remains qualitatively the same.

Let us call $u(t)$ the population of cells in the transit from ISC to EC at time t. Now we have, aside of ISC, two interdependent populations of cells: EC and u cells. The equations for case 1/ now read:

$$\frac{du}{dt} = \beta C \frac{\gamma}{E} I - \theta u \qquad (9)$$

$$\frac{dE}{dt} = \theta u - \alpha I. \qquad (10)$$

If we rewrite explicitly the above equations in terms of the lifetime τ of u cells, using equation (8) we are led to:

$$\begin{aligned} \frac{du}{dt} &= \beta C \frac{\gamma}{E} I - \frac{u}{\tau} \\ \frac{dE}{dt} &= \frac{u}{\tau} - \alpha I. \end{aligned}$$

The fixed point of the dynamical system defined by equations (9) and (10) is:

$$\begin{aligned} u^\star &= \frac{\alpha I}{\theta} \\ E^\star &= \beta C \frac{\gamma}{\alpha}. \end{aligned}$$

We observe that the predicted amount of EC is independent of the time τ needed for transiting from ISC to EC. In consequence, it remains invariant with respect to the previous simplified model.

The above approach assumed that the inhibition is happening at the level of differentiation. If one assumes case 2/, i.e., that the inhibition takes place at the level of proliferation, we are led to:

$$\frac{du}{dt} = \beta C \frac{\gamma}{E} I - \theta u - \alpha I \qquad (11)$$

$$\frac{dE}{dt} = \theta u. \qquad (12)$$

We notice that the dynamical system defined by equations (11) and (12) is obtained by just moving the term $-\alpha I$ to the first equation of the dynamical system defined by equations (9) and (10). Similar equations have been derived for the regulation of cortisol hormone levels [3]. The fixed point now reads:

$$\begin{aligned} u^\star &= 0 \\ E^\star &= \beta C \frac{\gamma}{\alpha}, \end{aligned}$$

i.e., the amount of EC remains the same, whereas cells in the intermediate state only appear temporarily as burst upon increases of the hormone. When the hormone levels stabilize, all newly produced cells end the differentiation process and become EC.

It remains to check the stability properties of the fixed points. To that end, we have to compute the Jacobian matrix at the fixed point and, since it is a 2D system, it is enough to check the signs of the trace and the determiner. Let us define $f(u,E) = \beta C \frac{\gamma}{E} I - \theta u$ and $g(u,E) = \theta u - \alpha I$. The Jacobian matrix of this 2D dynamical system is:

$$J = \begin{pmatrix} \frac{\partial f}{\partial u} & \frac{\partial f}{\partial E} \\ \frac{\partial g}{\partial u} & \frac{\partial g}{\partial E} \end{pmatrix} = \begin{pmatrix} -\theta & -\beta C \frac{\gamma}{E^2} I \\ \theta & 0 \end{pmatrix}.$$

It turns out that, if we define $f(u,E) = \beta C \frac{\gamma}{E} I - \theta u - \alpha I$ and $g(u,E) = \theta u$ the Jacobian matrix is the same. We now check the signs of the trace and the determiner at the fixed point:

$$\boldsymbol{tr}\begin{pmatrix} -\theta & -\beta C \frac{\gamma}{E^{\star 2}} I \\ \theta & 0 \end{pmatrix} < 0, \begin{vmatrix} -\theta & -\beta C \frac{\gamma}{E^{\star 2}} I \\ \theta & 0 \end{vmatrix} > 0,$$

a result easy to check because all the involved constants are positive. Given this combination of signs, $\boldsymbol{tr}(J) < 0$ and $|J| > 0$ we conclude that the fixed point is stable [4].

Observations on the generality of the results

The above computations have been performed by assuming that i) $c(0, \gamma/E)$ is a linear growing function of γ/E, ii) that the proliferative term in the dynamical equation (6) is linear as well with I –see equation (5)– and iii) that the inhibitory term α is constant and does not depend on γ/E.

The choice of linear and constant functions has been performed for the sake of simplicity. Let us briefly comment on the generality of the main result, namely, the existence of a stable fix point in E that shifts monotonously with γ/E as described in equation (7). We observe that the same qualitative results could be achieved by considering both dependencies described in i) and ii) just monotonously increasing functions, not necessarily linear.

The case of the inhibitory term α, commented in iii) can be as well generalized. Let us assume that $\alpha = \alpha(\gamma/E)$ and a generalized proliferation term $f(\gamma/E)$. Now, equation (6) reads:

$$\frac{dE}{dt} = If\left(\frac{\gamma}{E}\right) - I\alpha\left(\frac{\gamma}{E}\right).$$

In this context, what we require is that both the inhibitory term $\alpha(\gamma/E)$ and the proliferative term $f(\gamma/E)$ are, positive, non-decreasing functions with γ/E and that there exists a E^* by which:

$$f\left(\frac{\gamma}{E^*}\right) = \alpha\left(\frac{\gamma}{E^*}\right),$$

i.e., that there is a cross-over between the proliferative and inhibitory terms. In addition, we require that, if $E < E^*$, then:

$$f\left(\frac{\gamma}{E^*}\right) > \alpha\left(\frac{\gamma}{E^*}\right),$$

and that, if $E > E^*$, then:

$$f\left(\frac{\gamma}{E^*}\right) < \alpha\left(\frac{\gamma}{E^*}\right).$$

With these general properties, it follows naturally that:

$$\frac{d}{dE}\left(If\left(\frac{\gamma}{E}\right) - I\alpha\left(\frac{\gamma}{E}\right)\right)\Bigg|_{E^*} < 0,$$

and, therefore, that the fixed point is stable.

Blocking of cell differentiation: ISC Proliferation

In the previous modelling we considered that the amount of ISC is constant, since the division of an ISC leads to one ISC and one differentiated EC. Using the same line of reasoning, now we consider the case where such differentiation is blocked in a way that a division of a single ISC creates other two ISC. In this setting, the amount of EC will remain constant and the amount of ISC will eventually grow in time. Explicitly, we have that:

- 20HE inhibits the proliferation of ISC at rate α/ISC
- 20HE activates the production of Wg in the existing EC which, in turn, activates the production of ISC from proliferation of already existing ISC. The production of Wg depends on the hormone concentration (amount of hormone/cell number).

Note that, since the relative volume of ISC is negligible when compared to the volume of the EC, we can safely assume that the volume of the organ remains constant. As we did before, in Fig.S11A-B we provide a schematic way to understand the construction of the dynamic equations for this case.

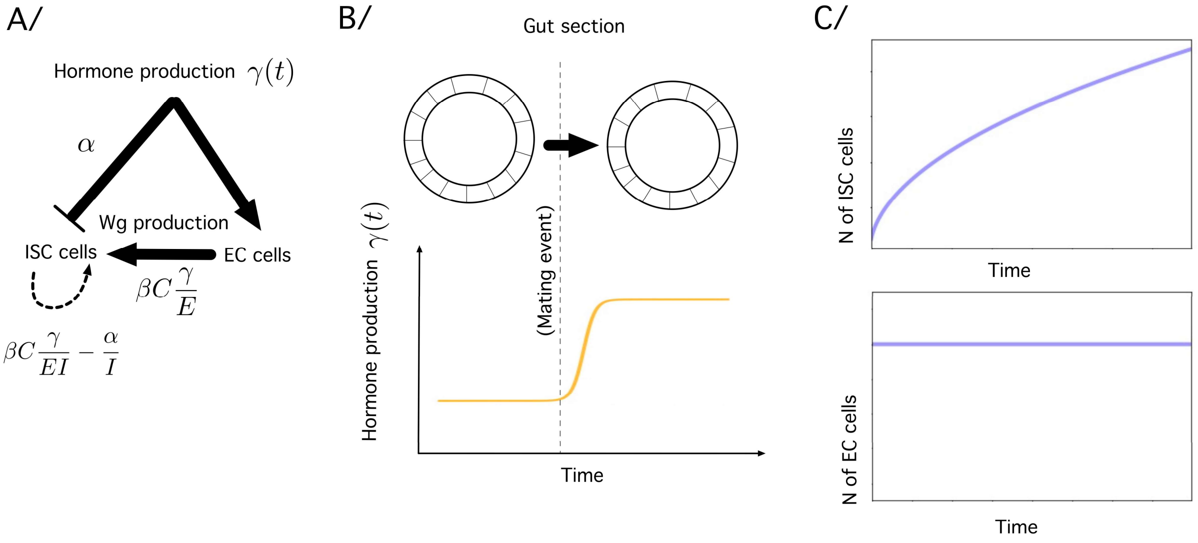

Fig.S11: Equation showing ISC numbers upon increased hormone levels and blocked EC differentiation

A/ Construction of the dynamical equations for the blocking of differentiation. B/ Contrary to the previous case, after a mating event there is no increase in midgut size (up) due to the increase of the hormone production (down), since the volume occupied by the ISC is negligible compared to the volume occupied by the EC. C/ (up) Evolution of ISC numbers when differentiation is blocked under constant hormone production, showing a square-root-like evolution in time. (bottom) the block of differentiation maintains a constant amount of EC.

Since both the amount of hormone and the amount of EC are constant, so will be the proliferative strength given by the amount of Wg. There is a positive constant K, that can be inferred from equation (5) as:

$$K \equiv \beta C \frac{\gamma}{E}.$$

This proliferative strength provided by the hormone level is assumed to be proportional to the Wg ligand produced by the EC, and will be shared by all the ISC. The same will happen with the inhibitor term, α. In consequence, the evolution of ISC number upon blocking of differentiation into EC will be given by:

$$\frac{dI}{dt} = \frac{K}{I} - \frac{\alpha}{I}.$$

As soon as $K > \alpha$, we expect to observe an unbounded growth of EC number–recall that here we are not considering other phenomena that could play a role! Indeed, considering that at $t = 0$ we have I_0 ISC, we expect to observe:

$$I(t) = \sqrt{2}\sqrt{(K-\alpha)t + I_0}.$$

Qualitatively, one expects, therefore:

$$I(t) \propto \sqrt{t}.$$

In Fig.S11C we have an example of this kind of growth.

References

[1] Berg, H. C. (1993) *Random walks in biology* Princeton University Press, Princeton

[2] Wartlick, O., Kicheva, A., González-Gaitán, M. (2009) Morphogen gradient formation. Cold Spring Harb. Perspect. Biol. 1(3):a001255

[3] Alon, U. (2024) *Systems Medicine. Physiological Circuits and the Dynamics of Disease* CRC Press (Boca Raton: FL)

[4] Strogatz, S. *Nonlinear Dynamics and Chaos : with Applications to Physics, Biology, Chemistry, and Engineering* Westview press (Boulder:CO)
